# Novel Type of Complicated Autosomal Dominant Hereditary Spastic Paraplegia Associated with Congenital Distal Arthrogryposis Type I

**DOI:** 10.3390/brainsci8070136

**Published:** 2018-07-19

**Authors:** Peter Hedera, Paolo Moretti, Jane Howard, Jiali Zhao

**Affiliations:** 1 Department of Neurology, Vanderbilt University Medical Center, Nashville, TN 37232, USA; jane.e.howard@vanderbilt.edu (J.H.); jiali.zhao@vanderbilt.edu (J.Z.); 2 Department of Neurology, University of Utah School of Medicine, Salt Lake City, UT 84132, USA; paolo.moretti@hsc.utah.edu; 3 George E. Wahlen Department of Veterans Administration Medical Center, Salt Lake City, UT 84148, USA; 4 Tennessee Valley Health Care System, Veterans Administration Medical Center, Nashville, TN 37212-2637, USA

**Keywords:** hereditary spastic paraplegia, complex phenotype, distal arthrogryposis

## Abstract

Hereditary spastic paraplegia (HSP) is one of the most genetically heterogeneous neurological disorders. HSP is classified as pure when only a spastic weakness of the lower extremities is present. Complex HSP comes with additional neurological or systemic abnormalities. Complex HSP with skeletal abnormalities is rare and mostly seen in autosomal recessive HSP. Autosomal dominant (AD) complex HSP with skeletal abnormalities are consistently seen only in SPG9 (spastic gait type 9). In this paper, we report a kindred condition with AD HSP among four living affected individuals who had progressive, adult onset spastic paraparesis that was associated with a distal arthrogryposis (DA) in every affected individual. They also had episodes of rhabdomyolysis without any clinical signs of myopathy. Exhaustive genetic analysis including targeted sequencing of known HSP and DA genes and whole exome sequencing did not identify the disease-causing gene. It excluded all known HSP and DA genes. We propose that this is a novel genetic type of complex AD HSP. Elucidation of a genetic cause of this type of HSP will further contribute to our understanding of axonal degeneration and skeletal abnormalities.

## 1. Introduction

Hereditary spastic paraplegia (HSP), which is also known as Strumpell-Lorraine disease, is a clinically and genetically heterogeneous group of neurodegenerative disorders characterized by progressive spasticity and weakness of the lower extremities [1,2,3]. Although spastic weakness of the legs is the hallmark of HSP, many patients also exhibit other neurological and non-neurological abnormalities. The presence of additional clinical problems is the basis for the phenotypic classification into a “pure” or uncomplicated HSP where spastic weakness of legs, bladder urgency, and sensory dysfunction attributable to dorsal columns dysfunction are the only features and complicated or complex HSP where additional neurological and non-neurological signs are present [1,4]. The most common clinical problems seen in complicated HSP are intellectual disabilities that can be either static or progressive cognitive impairments that lead to dementia, epilepsy, extrapyramidal signs with dystonia or Parkinsonism, more extensive ataxia than mere disruption of spinocerebellar tracts and includes nystagmus and cerebellar dysarthria, various degrees of leukoencephalopathy, retinopathy, deafness, optic nerve atrophy, hydrocephalus, partial or complete agenesis of the corpus callosum, polyneuropathy, and distal amyotrophy [1,2,3,4].

Even though it is now accepted that many patients with an apparently pure HSP commonly have additional neurologic problems including dementia in SPG4 (spastic gait 4) and polyneuropathy in several types of autosomal dominant (AD) HSP, this clinical classification remains a useful clinical concept [3,5,6]. Extra-neuronal manifestations of HSP such as cataracts, skin ichthiosis, or pigmented lesions, gastro-esophageal reflux with uncontrollable vomiting, and orthopedic abnormalities with short stature, scoliosis, congenital hip dislocation, and foot deformities may also contribute to our understanding of the pathogenesis of these disorders.

Troyer syndrome, which is caused by homozygous mutations of the *SPG20/spartin* gene, is a prototypical complicated HSP with skeletal abnormalities including short stature, foot deformities, and kyphoscoliosis. These are cardinal features of this entity [7,8]. Spartin also plays a role in the degradation of the epidermal growth factor receptor, which may account for impaired bone growth. Short stature with skeletal abnormalities and connective tissue abnormalities are also symptoms commonly seen with the *PNPLA6* gene-related group of disorders known as SPG39 [9,10].

Multiple congenital contractures affecting at least two different body segments are also known as arthrogryposis and they can be seen in more than 300 heterogeneous conditions [11]. Many times they may be caused by fetal akinesia even though typically neurological impairment in HSP is not sufficient to cause these secondary joint contractures in utero [12]. Multiple joint contractures can be seen in some forms of autosomal recessive (AR) HSP including SPG18 caused by mutations in the *ERLIN2* gene, SPG28 with biallelic DDHD1 (Phoshatidic acid-preferring phospholipase A1) mutations, and SPG43 with mutations in the *C19ORF12* gene among others [2,3].

Clinically isolated distal arthrogryposes are a group of AD disorders that mainly involve the distal parts of the hands and feet including pes equinovarus without a primary muscle disease or any other syndromic findings. The prototypic distal arthrogryposis is type 1, which is characterized largely by camptodactyly and clubfoot [11,12]. The shoulders and hips are less frequently affected. The degree of joint contractures is variable from mild to severe and the most severe form of hand arthrogryposis results in the clenched fists and ulnar deviation of the wrist and severe pes equinovarus in the foot.

Autosomal dominant distal arthrogryposes are typically caused by mutations in different sarcomeric proteins including β-tropomyosin (*TPM2*), fast troponin T (*TNNT3*), fast troponin I (*TNNI2*), embryonic myosin heavy chain (*MYH3*), fetal myosin heavy chain (*MYH8*), and slow myosin binding protein C (*MYBPC1*) [13,14]. Muscle pathology in distal arthrogryposis usually reveals only minor changes and only mutations in the *TNNI2*, which may have more prominent myopathic changes and an elevation of serum creatine kinase (CPK) [14].

In this paper, we report a kindred condition with AD HSP among four affected individuals who had progressive, adult onset spastic paraparesis that was associated with a congenital distal arthrogryposis in every affected individual and episodic rhabdomyolysis without any other signs of myopathy. Extensive genetic evaluation including whole exome sequencing did not identify any causative mutations. We propose that this is a novel type of complex AD HSP.

## 2. Materials and Methods

### 2.1. Clinical and Laboratory Studies

**Figure 1:**
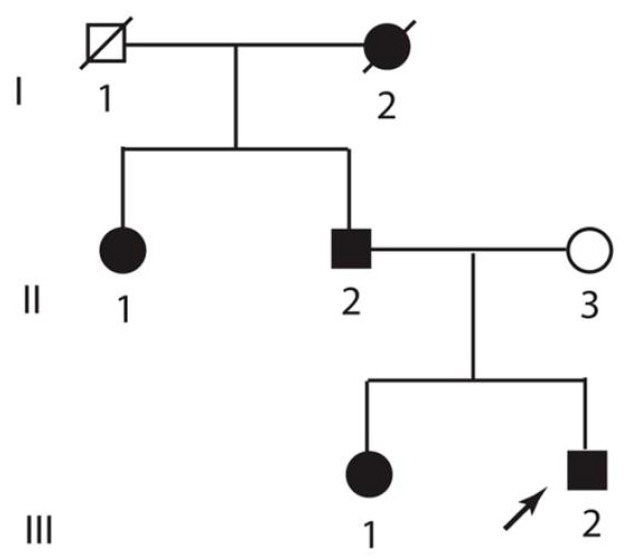
Pedigree of presented kindred: Roman numerals denote generation and Latin numerals denote individual subjects. The arrow indicates the proband.

We identified a small 2-generational kindred condition with four affected living individuals through a proband (subject III/2, Figure 1). All subjects consented to participate in this study, which was approved by the Institutional Review Board at Vanderbilt University (protocol Genetics of Neurologic Disorders #030029, approved on 10 April 2006). They underwent comprehensive neurological evaluation. Information about psychomotor development, age at symptom onset, and progression of disability was collected. Radiologic evaluation of hands and feet was also performed and the proband underwent additional radiologic evaluation with an MRI scan of the brain and the C spine as well as an electromyographic evaluation with nerve conduction studies. A muscle biopsy from the vastus lateralis was also performed on proband with frozen and formalin-fixed sections stained with hematoxylin and eosin, Gomori trichrome, ATPase (pH 9.4 and 4.2), and NADH (DPNH). A frozen muscle sample was also assayed on a myoglobinuria panel performed by the Robert Guthrie Biochemical Laboratory (Buffalo, NY, USA) with enzymatic activities of myoglobin phosphorylase, phosphorylase 1b kinase, phosphofructokinase 3-phosphoglycerate kinase, phosphoglyceratemutase, lactate dehydrogenase, and carnitine palmitoyiltransferase II (CPT2), which was measured and compared to normal values.

### 2.2. Genetic Analysis

The phenotype of each individual was determined as definitely affected or unaffected before genetic analysis. DNA was available from all affected living subjects and it was isolated using standard procedures. The first phase of genetic analysis was focused on the exclusion of candidate genes for AD HSP and AD distal arthrogryposis type 1. Coding exons of analyzed genes were amplified using the polymerase chain reaction with published or custom-designed intron-based primers and conditions. Polymerase chain reaction products were purified through Sephadex G-50 columns (Sigma, St Louis, MO, USA) and sequenced using an ABI PRISM dRhodamine Terminator Cycle Sequencing Ready Reaction and the ABI PRISM 3100 Genetic Analyzer (PE Applied Biosystems, Foster City, CA, USA), which was described previously [15]. Each exon was sequenced in both directions in two affected members of this family. Observed sequence changes were further analyzed for segregation with disease in every affected subject in the family. The SPG9 locus (before the causative gene was reported) was also genotyped and haplotypes were constructed for each family member. *Spast* (SPG4), *SPG3A* (atlastin 1), *NIPA1*, *REEP1*, and *KIF5A* genes were analyzed as candidate genes for HSP. β-tropomyosin (*TPM2*), fast troponin T (*TNNT3*), fast troponin I (*TNNI2*), embryonic myosin heavy chain (*MYH3*), fetal myosin heavy chain (*MYH8*), and the slow myosin binding protein C (*MYBPC1*) were included as candidate genes of distal arthrogryposis. Clinical genetic analysis also included an analysis of copy number variation (CNV) using the multiplex ligation-dependent robe amplification (MLPA) assay to detect possible deletions or duplication. This was performed using a commercial laboratory. Subjects III/1 and III/2 were analyzed.

The second stage of genetic analysis was performed with whole exome sequencing (WES) of three subjects (II/2, III/1, and III/2) using Illumina HiSeq 2500 platform (Illumina, San Diego, CA, USA). Genomic DNA was sheared to yield 100–450 base pair (bp) fragments, which was described before [16]. In-solution whole-exome capture and massively parallel sequencing was performed using the Agilent SureSelect^XT^ All Exon Kit 51 Mb (Agilent, Santa Clara, CA, USA). Enriched DNA fragments were sequenced on the Illumina's HiSeq 2500 platform, which were paired-end 100–125 bp reads. On average, more than 95% of exons were covered at >20×. The percentage of exome coverage was based on exons targeted by the 51 Mb All Exon v4 Kit (Agilent, Santa Clara, CA, USA), which incorporates the Consensus Coding Sequence (CCDS), the NCBI Reference Sequence (RefSeq), and GENCODE annotations. Sequence reads (FASTQ) from Illumina were mapped to the human reference genome (NCBI build 37.1).

Variants were annotated to RefSeq gene definitions using ANNOVAR software [17]. Damaging non-synonymous variation was defined as protein-altering substitutions predicted to be damaging by a consensus of at least three out of six prediction scores downloaded via dbNSFP (SIFT, Polyphen2 HDIV, LRT, MutationTaster, MutationAssessor, and FATHMM) [18,19,20,21,22]. Gene prioritizing was based on known genes causing HSP, arthrogryposis, genes expressed in the brain and muscle using the Center for Biotechnology Information (NCBI), Online Mendelian Inheritance in Man (OMIM), and literature searches. Putative deleterious variants were also checked for disease segregation in other affected family members. We sequenced these DNA segments including the subject II/1 when segregation with the phenotype was suggested using standard sequencing methods.

## 3. Results

### 3.1. Clinical Description

**Figure 2:**
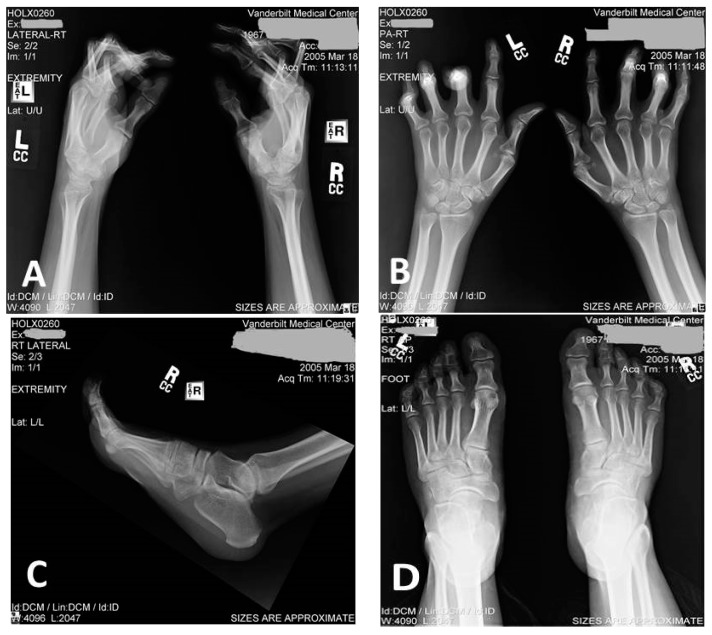
Radiograph of proband’s hands (**A** = lateral view; **B** = anterio-posterior (A/P view) and feet (**C** = lateral R foot and **D** = A/P both feet).

All included subjects (four living and one deceased) had a history of congenital distal arthrogryposis that was more prominent in the hands (Table 1). The severity of hand deformities was more pronounced in affected males with medially overlapping fingers, clenched fists, and ulnar deviation of fingers (Figure 2). Proband and his father required several corrective surgeries. Living affected females had a milder involvement of hand joints and they had camptodactyly of the third, fourth, and fifth fingers without signs of overlapping fingers. Deceased subject I/2 was described to have severe hand and feet deformities with bilateral club foot. However, her medical records were not available. She also developed progressive gait disorder in her 30s and, in her 50s, she was non-ambulatory. All affected individuals had a normal stature and no signs of other skeletal abnormalities.

Motor and psychosocial development was normal in every affected subject and they acquired all milestones without any delays. All affected individuals had gait with a tendency for tiptoeing and this was attributed to reduced dorsiflexion of ankles caused by feet deformities. This has resolved by five years of age and they did not have any signs of early spasticity and their gait was later reported as normal. First, neurological problems were reported in the third decade of life and were described as episodes of weakness and muscle pain including passing very dark urine. Medical records were available for subjects III/1 and III/2 and they had documented episodes of rhabdomyolysis with CPK elevations in the range of 20,000 U/L to 50,000 U/L. Some episodes were provoked by more strenuous activities but also unprovoked episodes were reported in every affected individual. The baseline CPK levels between the episodes of rhabdomyolysis were normal.

Changes in gait were reported during the third or fourth decade of life. At the ages of 30 years, 39 years, 27 years, and 32 years, subjects I/1, II/1, II/2, III/1, and III/2, respectively, were reported to have an onset of tripping and stiffness. They had signs of otherwise isolated spastic paraparesis with a reduced vibratory sensation. No signs of spasticity were present in the upper extremities. Their gait was spastic with a typical scissoring and no signs of ataxia. They had a tendency to walk on tiptoes and this was associated with reduced passive dorsoflexion in ankles. No other neurologic abnormalities were present and they all had a normal proximal strength and a mild distal weakness attributed to arthrogryposis.

Proband underwent additional clinical testing and his repeated electromyography with nerve conduction studies did not show any signs of myopathy or neuropathy. An MRI of the brain and C spine was unremarkable. A skeletal survey did not identify any other bone abnormalities other than hand and foot deformities. A muscle biopsy was also considered normal and he did not have any specific abnormalities with a normal, random pattern of fiber types and no evidence of group atrophy or fiber type grouping. There were no “ragged red” fibers, target fibers, or abnormal inclusions. An occasional and very mild variation in fiber sizes was observed, but this was considered non-specific. Enzymatic testing of the muscle tissue focusing on the myoglobinuria profile was also within normal limits. Baseline CPK levels were within normal values.

### 3.2. Genetic Analysis

Sequencing of candidate genes associated with AD HSP and AD artrogryposis did not reveal any disease-causing mutations. We were also able to exclude the SPG9 locus because of a random distribution of reconstructed haplotypes. CNV MLPA analysis did not detect any deletions or novel duplications that were considered pathogenic. WES identified 20,856 variants for subject II/2, 20,789, and 20,047 for subject II/3. 960 variants that were non-synonymous or potentially affected splice sites were selected for further analysis. No definite disease-causing mutations were identified in the kindred condition and we identified more than 40 putative non-coding and coding changes that would segregate with the disease (data not shown).

## 4. Discussion

HSP has emerged as one of the most genetically diverse syndromes with nearly 70 disease-causing genes identified so far [1,2]. The era of WES has accelerated the speed of discovery of new HSP genes. Additionally, several novel AR genes have been identified using this approach [23]. However, genetic etiology remains unknown in 38% of AD HSP cases and this is even higher for AR and apparently sporadic cases [24]. This may be caused by mutations in the non-coding regions. Additional genetic methods such as whole genome or RNA sequencing may be needed to identify the causes in these patients.

We propose that this type of HSP represents a novel type of AD complex HSP. Affected individuals exhibited congenital arthrogryposis, which was followed by the development of recurrent episodes of myoglobinuria without the evidence of myopathy. Later they experienced adult onset of a spastic gait. Even though the likelihood of coexisting separate conditions cannot be fully excluded without any proven unifying genetic cause, this is somewhat unlikely because we observed a complete co-segregation of these diverse phenotypic features in all analyzed patients. Furthermore, the likelihood that WES would not identify either of two or more causative genes, if one assumes a coexistence of several independent phenotypic features, is also quite low. The overlap of three different phenotypic features, which include arthrogryposis, spastic paraplegia, and recurrent rhabdomyolysis, may also suggest a contiguous gene syndrome caused by deletions of several genes. However, clinical analysis of copy number variation did not identify any large deletions that could account for this mechanism. An additional possibility is a biogenic inheritance, but the pattern of transmission in the kindred condition is more consistent with a straightforward AD mode. This would be only the third type of AD complex HSP where musculoskeletal abnormalities are a constant feature of the clinical phenotype.

Musculoskeletal problems are relatively rare in AD HSP [3]. Patients harboring mutations in the aldehyde dehydrogenase 18 family known as the member A1 (*ALDH18A1*) gene exhibit spastic paraplegia associated with short stature and bone dysplasia, gastroesophageal reflux, hiatal hernia, and cataracts [25,26]. This type of HSP is classified as SPG9 and additional acronyms have been suggested including CMNSS (cataracts with motor neuronopathy, short stature, and skeletal abnormalities) and SPACGR (spastic paraparesis with amyotrophy, cataracts, and gastroesophageal reflux). A single kindred with diffuse spasticity and multiple exostoses segregating as an autosomal dominant trait was also described, but the genetic cause remains unknown [27]. Another example of AD HSP with skeletal abnormalities is SPG33, which is caused by mutations in the *ZFYVE27/protrudin* gene [28]. Only one genetic family was identified so far (SPG33) and some patients had pes equinus. Pes equinus is a congenital foot deformity and it can be a part of the clubfoot abnormality spectrum with fixation of the foot in cavus and adductus (inclined inwards), varus (axially rotated outwards), and equinus (pointing downwards) positions with concomitant soft tissue abnormalities. However, it remains unclear if this is a consistent phenotypic feature of associated HSP. This is further complicated by the fact that the functional consequences of the *ZFYVE27/protrudin* mutation p.G191V have been challenged and it was suggested that it may actually represent a benign single nucleotide polymorphism [29].

Pathogenesis of HSP is very diverse but the abnormalities of intracellular transport have emerged as one of the cardinal mechanisms of distal axonal degeneration [30,31,32]. We excluded all known HSP genes including all kinesin molecules implicated in HSP. The coexistence of spastic paraparesis with the distal arthrogryposis and recurrent rhabdomyolysis would suggest that the possible underlying genetic defect is caused by a gene expressed in both the central nervous system and muscles. Members of the myosin superfamily are especially very plausible candidate genes because of their role in the synaptic transport and muscle integrity [33]. Our ongoing research efforts are also focused on these genes and the analysis of expression levels of these proteins.

## 5. Conclusions

We describe a novel type of AD complex HSP with skeletal abnormalities and episodes of rhabdomyolysis without any clinical evidence of myopathy. Our extensive genetic analysis including WES has not identified a causative gene but the additional analysis is ongoing. Identification of new kindreds with the same phenotype should facilitate the discovery of a genetic cause of this type of HSP.

## Figures and Tables

**Table 1:** Summary of phenotypic features in affected individuals.

Subject	Skeletal Abnormalities	Spastic Gait	Age of Onset of Paraparesis (Years)	Rhabdomyolysis
I/2	Both hands and feet with bilateral pes equinovarus	Yes, with wheelchair-dependency in her 50s	30s	Unknown
II/1	Mild hand deformities	Yes, required an assistive device in her 50s and wheelchair in her 60s	30	Yes (history of passing very dark urine)
II/2	Severe hand deformities, mild feet deformities	Yes, required assistive device in his 60s, wheelchair in his 70s	39	Yes (history of passing very dark urine)
III/1	Mild hand deformities	Yes, abnormal but independent gait at age 47 years	27	Yes (documented elevation of CPK and myoglobinuria)
III/2	Severe hand deformities, mild feet deformities	Yes, abnormal but independent gait at age of 51 years	32	Yes (documented elevation of CPK and myoglobinuria)

CPK = Creatine phosphokinase.
